# Inhibiting the Growth of 3D Brain Cancer Models with Bio-Coronated Liposomal Temozolomide

**DOI:** 10.3390/pharmaceutics13030378

**Published:** 2021-03-12

**Authors:** Giordano Perini, Francesca Giulimondi, Valentina Palmieri, Alberto Augello, Luca Digiacomo, Erica Quagliarini, Daniela Pozzi, Massimiliano Papi, Giulio Caracciolo

**Affiliations:** 1 Dipartimento di Neuroscienze, Università Cattolica del Sacro Cuore, Largo Francesco Vito 1, 00168 Rome, Italy; giordano.perini@unicatt.it (G.P.); valentina.palmieri@unicatt.it (V.P.); 2 Fondazione Policlinico Universitario A. Gemelli IRCSS, 00168 Rome, Italy; alberto.augello@unicatt.it; 3 Department of Molecular Medicine, Sapienza University of Rome, Viale Regina Elena 291, 00161 Rome, Italy; francesca.giulimondi@uniroma1.it (F.G.); luca.digiacomo@uniroma1.it (L.D.); daniela.pozzi@uniroma1.it (D.P.); 4 Istituto dei Sistemi Complessi, CNR, Via dei Taurini 19, 00185 Rome, Italy; 5 Department of Chemistry, Sapienza University of Rome, P.le A. Moro 5, 00185 Rome, Italy; erica.quagliarini@uniroma1.it

**Keywords:** biomolecular corona, nanomedicine, drug delivery, glioblastoma, temozolomide

## Abstract

Nanoparticles (NPs) have emerged as an effective means to deliver anticancer drugs into the brain. Among various forms of NPs, liposomal temozolomide (TMZ) is the drug-of-choice for the treatment and management of brain tumours, but its therapeutic benefit is suboptimal. Although many possible reasons may account for the compromised therapeutic efficacy, the inefficient tumour penetration of liposomal TMZ can be a vital obstacle. Recently, the protein corona, i.e., the layer of plasma proteins that surround NPs after exposure to human plasma, has emerged as an endogenous trigger that mostly controls their anticancer efficacy. Exposition of particular biomolecules from the corona referred to as protein corona fingerprints (PCFs) may facilitate interactions with specific receptors of target cells, thus, promoting efficient internalization. In this work, we have synthesized a set of four TMZ-encapsulating nanomedicines made of four cationic liposome (CL) formulations with systematic changes in lipid composition and physical−chemical properties. We have demonstrated that precoating liposomal TMZ with a protein corona made of human plasma proteins can increase drug penetration in a 3D brain cancer model derived from U87 human glioblastoma multiforme cell line leading to marked inhibition of tumour growth. On the other side, by fine-tuning corona composition we have also provided experimental evidence of a non-unique effect of the corona on the tumour growth for all the complexes investigated, thus, clarifying that certain PCFs (i.e., APO-B and APO-E) enable favoured interactions with specific receptors of brain cancer cells. Reported results open new perspectives into the development of corona-coated liposomal drugs with enhanced tumour penetration and antitumour efficacy.

## 1. Introduction

Brain tumor is a health and social issue of considerable importance. It causes about 7% of cancer-related deaths for those under the age of 70 and it is the second most common form of cancer (after leukemia) for children and teens [1]. Among primary brain cancers, glioblastoma (GMB) is the most common and lethal form [2]. Despite aggressive therapy, this tumor is characterized by frequent relapse [3]. Surgical resection, followed by radiation with simultaneous chemotherapy, as part of a combined modality approach, is among the most frequent current treatments. Yet, although recent advances in handling many solid tumors, the treatment of GBM remains weak with a median survival of 12–15 months [4]. Treatment limits derive from radiotherapy and chemotherapy resistance, side effects limiting treatments and, mostly, low drug concentration in the brain [5,6]. In fact, an arduous challenge in the management of brain tumor with drug administration is the drug tumor-targeting. As a matter of fact, to reach the brain, the drug needs to overcome the blood–brain barrier (BBB), a physical and electrostatic barrier that limits brain permeation of therapeutics [7,8]. Considering that, a special goal is increasing the bioavailability of traditional brain tumor chemotherapeutic drugs such as Temozolomide (TMZ), doxorubicin hydrochloride, irinotecan hydrochloride and vincristine sulfate [9,10,11,12,13]. Temozolomide (TMZ) is a chemotherapy drug that has been shown to improve average survival rate for people with some high-grade brain tumors. In clinical studies, temozolomide consistently demonstrates reproducible linear pharmacokinetics with approximately 100% p.o. bioavailability, noncumulative minimal myelosuppression that is rapidly reversible and activity against a variety of solid tumors in both children and adults [14]. However, in aggressive tumors, TMZ treatment is rarely successfully curative, because of tumor’s high resistance to therapy and low drug bioavailability in tumor tissue. [15] Thus, new alternatives are necessary, including tumor cells-targeted approaches through the use of directed vectors that are able to be transported across the BBB and accumulate in the target tissue. One approach towards increasing drug accumulation in cells, is to escape from their efflux mechanism by encapsulating drugs into nano-scaled vehicles [16], e.g., poly(D,L-lactide-co-glycolide) (PLGA) [17], chitosan nanogel [18], iron oxide nanoparticles [19,20] and lipid nanoparticles [21]. Indeed, one of the most promising and versatile strategies makes use of nanoparticles (NPs) acting as drug delivery systems since they provide both protection to therapeutic agents and efficient delivery across the BBB [22]. Among different nano-vehicles, liposomes are considered potent nanocarriers systems for controlled drug release [22,23]. This is mainly due to their non-toxic nature, biocompatibility, size, specificity and membrane permeability. For instance, Patel and Parikh have compared the anti-cancer efficiency of free TMZ and TMZ loaded in Hydro Soy L-α-phosphatidylcholine (HSPC) liposome for the GBM management through cell culture technique [22]. Their results suggested that liposomal formulation enhances the availability of drug at the specific site and reduces the dose-related toxicity of chemotherapy. However, despite the success of liposomal formulations in vivo, their translation into clinic has progressed more slowly. Indeed, these delivery systems are prone to elimination from the bloodstream, limiting their therapeutic efficacy. This clearance may be due to several factors including opsonization of plasma proteins or uptake by fixed macrophages. Thus, to overcome this limitations, newer generation of liposomes have employed a combination of sterically stabilized and ligand-targeted liposomes to enhance their circulation times in blood and assure a site-specific release. For example, in a recent study, it has been demonstrated that multi-targeting liposome based on glucose and biotin showed a more consistent cellular uptake of glioma cells than uncoated liposomes [24]. Yet, in the clinic, most of these data still remain unavailable for many nanomedicines. This is principally owed to the wrong assumption that liposomes will directly interact with cell surface once administrated. This is principally owed to the wrong assumption that liposomes will accumulate at the target site once administrated [25]. In the last decade, on average, less than 1% of the dispensed dosage of nanomaterials has been found within a solid tumor [26]. This poor accumulation ability has harmful influence on the clinical translation of nanomaterials for human use with respect to cost, toxicity and therapeutic outcome. After ten years of intense investigation, we now know that the poor translation of liposomes from benchtop to patient’s bedside is mainly due to our lack of knowledge of the bionano interactions, i.e., the interactions between nanosized liposomes and biological systems [27,28]. In fact, a common factor that is often ignored when dealing with the synthesis of drug delivery systems is testing their behavior in biological fluids [29]. Although an efficient translation of a drug delivery system relies on rigorous control over its physicochemical characteristic such as size, charge, morphology etc., these parameters are often altered upon contact with biological media. Following intravenous administration, liposomes interact with biological molecules present in blood which adsorb on their surface forming a “corona” [30,31,32]. This biomolecular corona (BC) alters the synthetic identity of the nanosystems conferring a new identity that mostly controls their biological activity (e.g., blood residency, biodistribution, immune system recognition, cell binding and intracellular fate) [27,33,34]. Furthermore, the exposition of particular biomolecules from the corona may facilitate interactions with specific receptors [35,36,37]. Currently, it is known that liposomes interact and bind to receptors at the BBB level [38]. Thus, it is maturing the idea that BC-based nano-delivery systems could be suitable for innovative treatments of brain-related diseases [39,40]. Consequently, to make the most of these novel aspects and generate an efficient drug delivery system for brain-targeting, it is essential to investigate whether the BC supports liposome-based brain targeting [41]. As a step to clarify this matter, this work was aimed at exploring the effect of BC on the drug penetration and anticancer activity of liposomal TMZ. We tackled this issue by employing four TMZ-encapsulating cationic liposomal (CL) formulations made of binary combinations of the cationic lipids 1,2-dioleoyl-3-trimethylammonium-propane (DOTAP) and 3(-[N-(N′,N′-dimethylaminoethane)-carbamoyl]-cholesterol (DC-Chol) and neutral lipids dioleoylphosphatidylethanolamine (DOPE) and cholesterol (Table 1). This formulation was chosen according to previous findings as it exhibits unusual endosomal escape that results in high performances and potential applicability in difficult-to-transfect cells [42,43,44,45].

Research on brain cancer drug response has historically been performed using commercially available 2D cell cultures that poorly predict in vivo cellular responses. In recent years, 3D cell culture techniques have been largely investigated and became a suitable alternative to traditional cell culture methods [1,46,47]. GBM tumor growth develops around three different areas, a proliferative outer region, a hypoxic core and a permeable vasculature. Currently, it has become apparent that 3D cultures reproduce more faithfully GBM features, from its spatial distribution to its interaction with the surrounding microenvironment [48]. Furthermore, 3D cultures are generally more resistant to chemotherapy [49]. Therefore, here we tested TMZ-loaded CLs on a 3D brain cancer model derived from U87 human glioblastoma multiforme cell line.

## 2. Materials and Methods

### 2.1. Preparation of TMZ-Loaded CLs

1,2-Dioleoyl-3-trimethylammonium-propane (DOTAP), (3-[N-(N′,N′-dimetylaminoethane)-carbamoyl])-cholesterol (DC-Chol), dioleoylphosphatidylcholine (DOPC) and dioleoylphosphatidylethanolamine (DOPE) were purchased from Avanti Polar Lipids (Alabaster, AL, USA). Cationic lipids were used in accordance with standard procedures [50] by dissolving appropriate amounts of lipids at ϕ = neutral lipid/total lipid (mol/mol) = 0.5. The encapsulation of Temozolomide (TMZ) into cationic liposomes (CLs) was performed through the dehydration-rehydration method by adding 3 mg TMZ to lipids in molar ratio 1:1 and dissolving the whole mixture in 1 mL of chloroform and 0.2 mL of methanol [51]. The mixture was placed on a rotary evaporator for 4 h at 65° to produce the film layer. After rehydration with 2.5 mL of PBS, the solution was extruded 20 times by means of a 0.1 μm polycarbonate filter with the Avanti Mini-Extruder (Avanti Polar Lipids, Alabaster, AL, USA) then, subjected to centrifugal filtration with Amicon Ultra-2 mL centrifugal filters (Merck Millipore, Darmstadt, Germany). Finally, the obtained TMZ-loaded CLs were incubated with human plasma (HP) for 1h at 37 °C to form the TMZ-loaded CL biomolecular corona (BC) complexes.

### 2.2. UV-Vis Spectra Measurements

The TMZ encapsulation efficiency (EE) and drug loading content (DLC) were measured by separating free TMZ from the TMZ Cls using Vivaspin 500 (5 kDa MWCO, GE Healthcare) and performing absorbance measurement of both free and encapsulated TMZ with Jasco V-630 spectrophotometer. Then, we obtained the concentration of TMZ by using the Lambert–Beer law to the 330 nm absorption peak and we correlated the measured concentration to the sample volume to obtain the absolute amount of free and encapsulated TMZ. Thus, EE and DLC were calculated through Equations (1) and (2) respectively:

EE = 100 × (mass of the drug in liposome)/(initial mass of the drug used)(1)

DLC = 100 × (mass of encapsulated drug)/(mass of liposome)(2)

### 2.3. Size and Zeta Potential Experiments

Size and zeta potential measurements of TMZ-loaded CLs and TMZ-loaded CLs-BC complexes were performed with a Zetasizer Nano ZS90 (Malvern Panalytical, Malvern, UK) at 25 °C. All samples were firstly diluted 1:100 with distilled and data were expressed as mean ± standard deviation of three replicates. As excess HP was not removed from the suspension of biocoronated CLs, size and zeta-potential measurements refer to the mixture BC liposomes and HP.

### 2.4. Cell Culture

U87 human glioblastoma cells were purchased from the American Type Culture Collection (ATTC, Manassas, VA, USA). Cells were maintained in Dulbecco’s modified Eagle’s medium (Sigma-Aldrich, St. Louis, MO, USA) supplemented with 10% fetal bovine serum (FBS, EuroClone), 2% penicillin-streptomycin (Sigma-Aldrich) and 2% L-glutamine (Sigma-Aldrich). Cells were cultivated in T75 flasks and kept at 37 °C in 5% CO_2_ humidity.

### 2.5. Spheroid Preparation and Drugs Administration

U87 human glioblastoma cells were seeded on 96-well, round bottom, ultra-low attachment plates (Corning, Corning, NY, USA) at a density of 0.5 × 10^5^ cells/ mL. The multiwell was centrifuged at 300 g for 3 min to ensure the confluence of cells to the centre of the wells. The so-formed single spheroids were incubated at 37 °C in 5% CO_2_ humidity for 3 days before further treatments. CLs containing TMZ were administered to spheroids at a final concentration of 0.5 mg/mL in two different conditions: pre-incubated with human plasma (Sigma, 1 h at 37 °C), to form protein corona, or without incubation. TMZ alone was administered to spheroids at 0.5 and 1 mg/mL. Control spheroids were used to compare results for both conditions, by administering human plasma or PBS respectively.

### 2.6. Spheroid Size and Cell Viability Measurements

After administration, spheroids were regularly imaged for 14 days at 4× magnification with Cytation3 Cell Imaging Multi-Mode Reader (BioTek, Winooski, VT, USA), by fixing focal height at 2455 μm and by performing auto-correction of the white balance for each well. Size analysis was carried out with ImageJ software [46]. Briefly, spheroid images were converted to 8-bit. A mask was created and the area of each spheroid was measured. Data were normalized by the initial volume (day 0) of each spheroid. After the time-course experiment, cell viability was assessed by the CellTiter-Glo^®^ Luminescent Cell Viability Assay (Promega, Madison, WI, USA). Results were normalized by respective control spheroids. Pristine liposomes (i.e., in the absence of the biomolecular corona) did not show any toxicity and cell viability was around 100% for all the four formulations.

## 3. Results and Discussion

TMZ encapsulation efficiency (defined as (mass of the drug in liposome)/(initial mass of the drug used) and the correspondent drug loading content (defined as (mass of encapsulated drug)/(mass of liposome)) of CLs were obtained by UV-Vis spectra analyses and the results are reported in Table 1. As shown, three out of four formulations exhibited EE values higher than 60% with CL2 that reached the highest one equal to 77.7 ± 5.1%. Then, TMZ-loaded CLs were incubated with human plasma (HP) for 1 h at 37 °C to form the TMZ-loaded CL-BC complexes.

**Figure 1:**
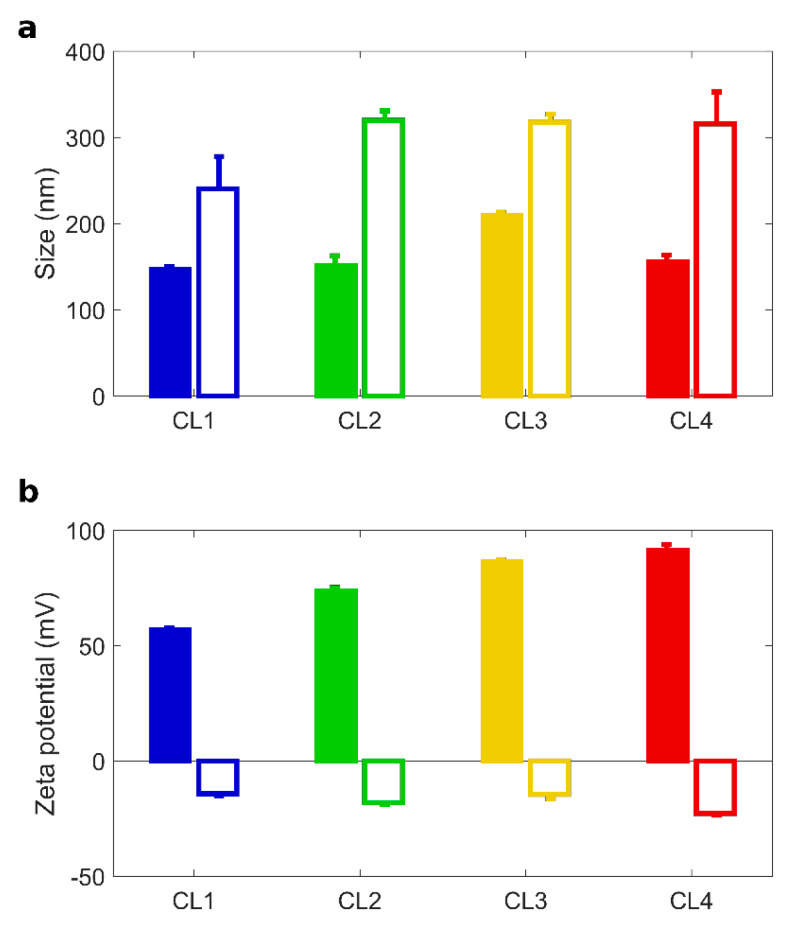
Size and zeta potential of TMZ-loaded CLs with and without BC. Size (**a**) and zeta-potential (**b**) of Temozolomide (TMZ)-loaded CLs (full histograms) and TMZ-loaded CL biomolecular corona complexes (empty histograms): CL1 (DOTAP/cholesterol), CL2 (DOTAP/DOPE), CL3 (DC Chol/DOPE), CL4 (DC-Chol/cholesterol).

A thorough characterization of CLs and CL-BC complexes is summarized in Table 2 and Figure 1. Dynamic Light Scattering and Electrophoretic Light Scattering measurements provided size and zeta potential of the investigated systems. TMZ-CLs were small in size (hydrodynamic diameter between 100 and 200 nm) and positively charged (zeta potential between 50 and 90 mV). Statistical differences between the four formulations in regard to all parameters mentioned in Table 2 were evaluated (Supporting Information).

**Figure 2:**
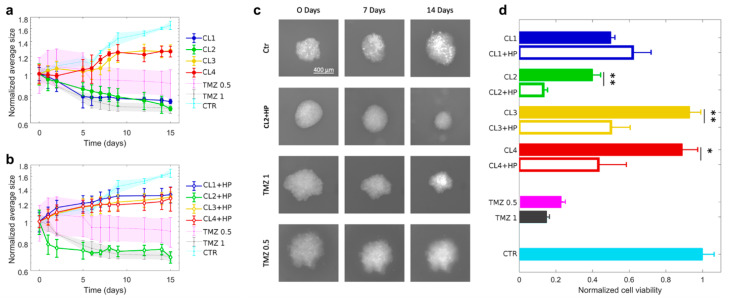
Glioblastoma spheroids treated with TMZ-loaded CLs with and without BC. (**a**) Spheroid growth in time expressed in terms of normalized average size after the treatment with TMZ-loaded CLs and (**b**) TMZ-loaded CLs incubated with human plasma (HP). (**c**) Representative images at 0, 7 and 14 days of glioblastoma spheroids after the treatment with control solution, TMZ-loaded CL2 incubated with human plasma (HP) and free TMZ at two different concentration (0.5 mg/mL and 1 mg/mL). (**d**) Cell viability of spheroids after two weeks of treatment with CLs (filled histograms) and CLs incubated with HP (empty histograms). CL1 (DOTAP/cholesterol), CL2 (DOTAP/DOPE), CL3 (DC Chol/DOPE), CL4 (DC-Chol/cholesterol). Statistical significance was evaluated by student’s test with respect to CLs incubated with HP. ** *p* < 0.05, * *p* < 0.001, no asterisk means not significant.

After 1 h incubation with HP, TMZ-CLs with BC were bigger in size than their counterparts (Figure 1a). The correspondent Polydispersity Indexes (PdI in Table 2) indicate that bare TMZ-CLs were homogenous in size, but TMZ-CLs with BC exhibited wider size distributions. These findings are most likely due to the formation of a thick protein layer at the particle surface leading to aggregation [41]. The inversion of the zeta potential is also caused by protein binding, as most plasma proteins exhibit negative charges at physiological pH. After preparation and chemical-physical characterization, TMZ-CLs with and without BC were diluted to have a final TMZ concentration of 0.5 mg/mL and administered to 3D spheroids [52]. Anticancer activity against GBM spheroids was evaluated by spheroids size distribution. Area of spheroids was monitored using digital microscopy to assess changes in spheroid size due to cell death and destruction of the spheroid architecture. Figure 2a reported the time course (from 0 to 14 days) of changes in tumor size, expressed as normalized average area, induced by the treatment with TMZ-loaded CLs, free TMZ at two different concentrations (0.5 and 1.0 mg/mL) and untreated spheroids as controls.

As depicted, after two weeks normalized average areas of spheroids, treated with TMZ-loaded CL1 and CL2, strongly reduces, respectively up to 75% and 69% with respect to their initial size. By contrast, the treatment with TMZ-loaded CL3 and CL4 seems not to affect cell proliferation. The different trends of the four CLs complexes could be mostly attributed to two possible factors, i.e., non-specific contributions related to the different physical-chemical properties of each complex or specific mechanisms associated to the different complex’s chemical compositions, on which mainly depends the molecular recognition at cell membrane level [53,54]. The first factor can be excluded since all complexes exhibited roughly the same diameter and surface charge (Table 2). Consequently, lipid composition is expected to play a role in activating specific endocytic pathways [54]. Indeed, CL1 and CL2 are both composed of 50% of the cationic lipid DOTAP while CL3 and CL4 of 50% of the cationic DC-Chol. The different composition seems to be the main factor in regulating cell recognition with the result that the presence of DOTAP in TMZ-CL1 and TMZ-CL2 led to a higher internalization and a resultant inhibition of cell growth. Next, since the purpose of the present study was to evaluate the impact of BC on tumor growth, we administrated CLs with BC to the GBM spheroids and measured the corresponding size changes in time (Figure 2b). It is evident that spheroids treated with TMZ-loaded CL2 with BC abruptly reduced in size already after two days. Thus, the presence of BC would seem to promote the complex’ cellular uptake. After two weeks the CL2 complexes with BC reduced spheroid growth with an effect like twice free TMZ (1 mg/mL). On the contrary, the effect of BC on the other formulations resulted irrelevant, despite the BC contributions in terms of size and surface charge was similar to all the complexes. According to recent literature [55], this inequality could be ascribed to a different BC composition that can either promote or inhibit cellular recognition. It was previously demonstrated that CL2 complexes are enriched of typical protein corona fingerprints (PCFs) (i.e., Vitronectin, APOA1, APOA2, APOB, APOC2, Ig heavy chain V−III region BRO, vitamin K-dependent protein and Integrin beta3) that trigger selective association with cancer cells leading to [56,57]. Among identified PCFs, Vitronectin binds to αvβ3 integrins, also known as the vitronectin receptor and was exploited to target cancer cells over-expressing αvβ3 integrins [37]. This is a point of great general interest, as αvβ3 integrins are overexpressed on U-87 cell line [58]. Apolipoproteins bind specific lipoprotein receptors, including low-density lipoprotein receptor (LDLR) and scavenger receptor class B type I (SR-BI). LDLR mediates the endocytosis of cholesterol-rich LDL, whereas SR-BI is a high-density lipoprotein (HDL) receptor that promotes cell internalization of cholesterol esters from circulating lipoproteins. Notably, both LDLR and SR-BI are upregulated in human gliomas and play an important role in the delivery and accumulation of cargos into human U-87 cells [59,60]. Importantly, it was not possible to measure spheroids treated with human plasma alone, since it caused spheroid disaggregation and invasion, as it has already been reported in literature [61]. For a complete view, in Figure 2c we reported the microscopy images of GBM spheroid size evolution from 0 to 7 and 14 days after administration with TMZ loaded CL2 with BC complexes in comparison with free TMZ and untreated spheroids. As a confirmation of previous findings, the images manifested the similarity between the size of spheroids treated with BC-CL2 complexes and free TMZ (1 mg/mL). According to these results, it is clear how the presence of BC does not have a unique effect on tumor growth. Thus, to have an exhaustive understanding of its influence on tumor activity, after two weeks we performed cell viability measurements on spheroids treated with TMZ-CLs with and without BC (Figure 2d). Except for TMZ-CL1 where the presence of BC seems not to significantly affect the viability compared to the counterpart without BC (respectively 0.62 ± 0.09 and 0.5 ± 0.01), for the other three complexes the reduction of cell viability is strongly stressed by the presence of BC. In particular, CL2 both with and without previous incubation with HP, exerted a significant reduction up to 0.13 ± 0.01 and 0.40 ± 0.03, respectively, similarly to free TMZ at 1 mg/mL (0.15 ± 0.003). By comparing tumor growth analysis and viability assay it is possible to draw some conclusions. Notably, except for CL2 complexes where BC displayed a remarkable impact both in size tumor decrease and cell viability, for the other cases there is a discrepancy between tumor growth trends and viability results. Specifically, BC, in most cases, did not exhibit an inhibitory effect on spherical growth but, on the other hand, had a significant impact on decreasing cell viability. A striking example is represented by TMZ-CL4 where the presence of BC had an irrelevant effect on spheroid size but not on cell viability that is reduced up to 0.43 ± 0.14. This divergence could depend on different aspects specifically related to the 3D cultures behavior. In the literature, several works correlate viability assays to the size growth trends of 3D cultures [49,62]. When dealing with a 3D culture growth analysis, it must be considered factors as cell density of the spheroid and cell cohesion. These two factors together are usually used to get a better estimate of the effective spheroid volume [63]. A decrease in cell adhesion corresponds to a decrease in density and an increase in spheroid volume. How does this relate to cytotoxicity? When cells begin to die, the ones that compose the peripheral region of the spheroid adhere less to each other, this phenomenon leads to an initial increase in the spheroid volume. After that, there is a consequential dissociation of the peripheral cells that cause a volume decrease. Thus, it is not always true that a decrease in cell viability is related to a decrease in spheroid area. To have a more reliable correlation between viability and spheroid growth, cell cohesion assays and density measurements should be developed in parallel. In conclusion, the effect of BC on the anticancer activity of TMZ-loaded CLs in a GBM spheroid culture was evaluated by means of spheroid size trends and cell viability assay. The results indicated a non-unique effect of the corona for all the complexes, especially regarding the tumor growth trends. An outstanding result was reported by TMZ-CL2 formulation with BC, that caused a notable reduction of tumor size in line with a considerable decrease of cell viability. This finding demonstrated that the exploitation of BC could be a helpful strategy to perform targeting nanodevice able to overcome the BBB and improve anticancer efficacy. However, further investigations are needed to better understand the mechanism behind cellular uptake in GBM spheroids’ cultures.

## 4. Conclusions

BC on the anticancer activity of TMZ-loaded CLs in a GBM spheroid culture was evaluated by means of spheroid size trends and cell viability assay. The results indicated a non-unique effect of the corona for all the complexes, especially regarding the tumor growth trends. An outstanding result was reported by TMZ-CL2 formulation with BC, that caused a notable reduction of tumor size in line with a considerable decrease of cell viability. This finding demonstrated that the exploitation of BC could be a helpful strategy to perform targeting nanodevice able to target brain cancer cells and improve anticancer efficacy.

## Figures and Tables

**Table 1:** Lipid compositions of TMZ-loaded CLs.

Sample Name	DOTAP (mol %)	DC-Chol (mol %)	DOPE (mol %)	Chol (mol %)
CL1	50	0	0	50
CL2	50	0	50	0
CL3	0	50	50	0
CL4	0	50	0	50

**Table 2:** Chemical-physical characterization of TMZ-loaded CLs before and after incubation with human plasma (HP). CL1 (DOTAP/cholesterol), CL2 (DOTAP/DOPE), CL3 (DC Chol/DOPE), CL4 (DC-Chol/cholesterol). Zeta deviation is intended as the width distribution as reported by the Malvern Zetasizer software.

	CL1	CL2	CL3	CL4
Size (nm)	147 ± 3	151 ± 11	209 ± 4	156 ± 8
Size upon exposure to HP (nm)	240 ± 37	319 ± 11	318 ± 9	316 ± 37
PdI	0.095 ± 0.013	0.164 ± 0.037	0.289 ± 0.020	0.206 ± 0.116
PdI upon exposure to HP	0.590 ± 0.049	0.453 ± 0.019	0.465 ± 0.030	0.692 ± 0.195
Zeta potential (mV)	56.8 ± 1.0	73.8 ± 1.4	86.4 ± 0.9	91.4 ± 0.8
Zeta potential upon exposure to HP (mV)	−14.2 ± 0.8	−18.1 ± 0.8	−14.5 ± 1.8	−22.8 ± 0.8
Zeta deviation (mV)	10.2 ± 0.5	11.8 ± 0.3	11.8 ± 0.5	12.1 ± 0.7
Zeta deviation upon exposure to HP (mV)	3.9 ± 0.2	4.9 ± 0.5	4.7 ± 1.0	4.0 ± 0.2
Encapsulation Efficiency	32.5 ± 1.2%	77.7 ± 5.1%	65.3 ± 4.3%	73.5 ± 3.4%
Drug Loading Content	14.9 ± 0.9%	23.9 ± 3.1%	22.9 ± 2.7%	31.7 ± 1.5%

## Data Availability

The datasets generated during and/or analyzed during the current study are available from the corresponding authors (M.P. and G.C.) on reasonable request.
